# The role of invasive and non-invasive imaging technologies and calcium modification therapies in the evaluation and management of coronary artery calcifications

**DOI:** 10.3389/fcvm.2023.1133510

**Published:** 2023-04-06

**Authors:** Samuel B. Wopperer, Rafail Kotronias, Federico Marin, Stefano Benenati, Francesco Della Mora, Leonardo Portolan, Adrian P. Banning, Giovanni Luigi De Maria

**Affiliations:** ^1^ Department of Cardiovascular Medicine, Mayo Clinic, Rochester, MN, United States; ^2^ Oxford Heart Centre, John Radcliffe Hospital, Oxford University Hospitals, NHS Foundation Trust, Oxford, United Kingdom; ^3^ Division of Cardiology, Department of Medicine, University of Verona, Verona, Italy

**Keywords:** non-invasive, invasive, coronary, calcium, imaging

## Abstract

The treatment of coronary artery disease (CAD) has advanced significantly in recent years due to improvements in medical therapy and percutaneous or surgical revascularization. However, a persistent obstacle in the percutaneous management of CAD is coronary artery calcification (CAC), which portends to higher rates of procedural challenges, post-intervention complications, and overall poor prognosis. With the advent of novel multimodality imaging technologies spanning from intravascular ultrasound to optical coherence tomography to coronary computed tomography angiography combined with advances in calcium debulking and modification techniques, CACs are now targets for intervention with growing success. This review will summarize the most recent developments in the diagnosis and characterization of CAC, offer a comparison of the aforementioned imaging technologies including which ones are most suitable for specific clinical presentations, and review the CAC modifying therapies currently available.

## Introduction

The presence of coronary artery calcification (CAC) continues to be an obstacle in the percutaneous management of coronary artery disease (CAD). In a pooled cohort analysis of the ACUITY (Acute Catheterization and Urgent Intervention Triage Strategy) and HORIZONS-AMI (Harmonizing Outcomes With Revascularization and Stents in Acute Myocardial Infarction) trials consisting of 6,855 patients presenting with acute coronary syndrome (ACS), it was found that patients with moderate or severe target lesion calcifications on coronary angiography were more likely to experience definite stent thrombosis and ischemia-driven target lesion revascularization at rates of 62% and 44%, respectively, as well as cardiac death within one year as compared to those with no or mild calcified coronary disease (1). This finding has been recently corroborated in a meta-analysis by Guedeney et al. in which 19,833 patients presenting with ACS as well as stable CAD were stratified by the severity of the target lesion calcification and use of a first- or second-generation drug-eluting stent (DES) and followed up to five years. Again, patients with moderate or severe CAC were more likely to experience target-lesion failure, ischemia-driven target lesion revascularization, and stent thrombosis as well as major adverse cardiovascular events. Patients treated with a second-generation DES had slightly lower event rates, indicating that the second-generation DES may help mitigate but do not solve the problem of CAC (2). More recently, a pooled analysis from the ISAR4-TEST and ISAR-5 studies has confirmed a clear association between significant calcification and poor outcomes at 10-year follow-up also in patients treated with second generation DES and irrespective of the DES-polymer coating strategy (i.e., permanent polymer vs. bioresorbable polymer vs. no-polymer) (3).

The purpose of this article is to offer a framework for understanding the characteristics that are prognostically significant in CAC, dissect in detail the properties and potentials of various imaging modalities to define CAC, and lastly, provide a brief overview of the currently available techniques to modify CAC. This manuscript aims to offer a careful and balanced review of the available evidence of both the diagnostic and therapeutic pathways though with the understanding that the studies included are not necessarily directly comparable and that there is no currently available universal reference standard for how CAC should be diagnosed and treated.

## Coronary artery calcification and poor prognosis: why?

The most important factor that determines short- and long-term percutaneous coronary intervention (PCI) success is the final minimum stent area (4, 5). CAC leads to stent failure *via* two general mechanisms. First, CAC leads to a decrease in vessel compliance (“distensibility”), which prevents full stent expansion and apposition to the coronary wall. Calcified atheromas have indeed proven to be up to 4–5 times less compliant than lipidic or fibrotic atheromas in studies completed nearly three decades ago (6). Second, the presence of CAC and subsequent vessel rigidity make delivery of equipment challenging. Difficult stent trackability, especially when coronary calcium is combined with vessel tortuosity, can lead to damage of the stent platform and/or polymer during repeated attempts to overcome a calcified segment. Electronic microscopy has shown that this can lead to phenomena of cracking, ridging, webbing, and peeling-off of the polymer. Damage of stent platform and polymer can interfere with the kinetics of drug elution and potentially cause long-term stent failure (6, 7).

However, it is not simply the presence of CAC but also its distribution within the coronary artery that can affect the final stent result. Initial work by Mintz et al. involving 1,155 native vessels analyzed by coronary angiography and intravascular ultrasound (IVUS) determined the following key characteristics of calcium burden:

1. Eccentricity of calcium (e.g. extent of circumferential involvement)—measured by the arc of calcium
2. Thickness of calcium—measured as the distance between the adluminal profile and the abluminal profile of the calcified component
3. Depth of the calcified plaque component within the vessel wall—measured as the distance of the adluminal profile from lumen contour
4. Longitudinal extension (e.g., length) of the calcified component along the course of the artery—measured as the distance from beginning to end of longitudinal calcium involvement (8).

**Figure 1:**
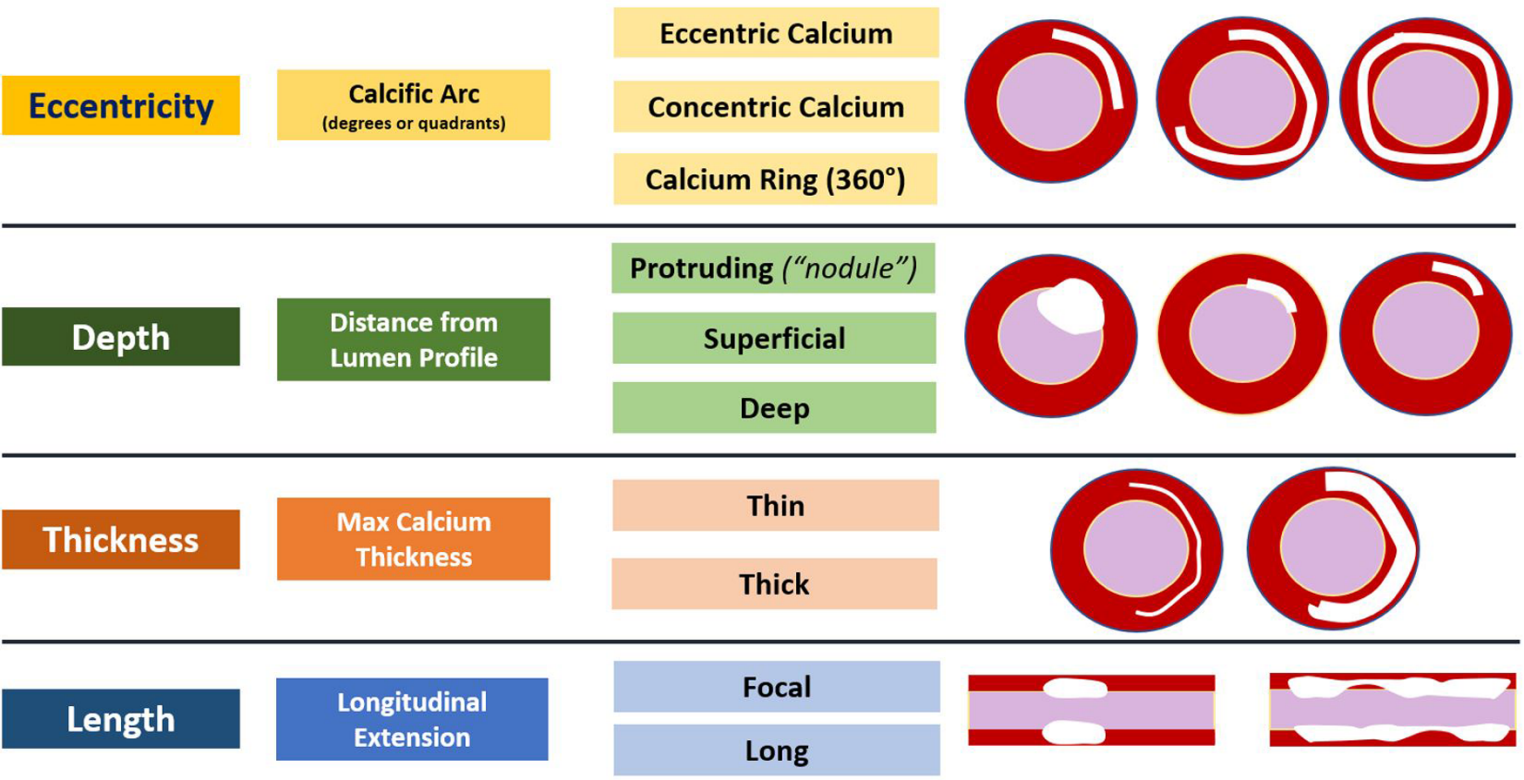
Summary of the variables that predict stent under-expansion and malposition as well as diagrammatic representations.

Using this as a foundation, subsequent work established that the presence and severity of these variables are also predictors of stent under-expansion and malposition (9). Diagrammatic representations of each variable are summarized (Figure 1).

Notably, patients may present with different combinations of these variables, and the key to optimal CAC preparation is in identifying which pattern is present. Once this is known, a calcium modifying therapy that is most suitable for that particular pattern can be chosen to alter CAC compliance and to allow for full balloon inflation and stent expansion. With the advent of advanced multimodality imaging including IVUS, optical coherence tomography (OCT), and coronary computed tomography angiography (CCTA), a full assessment of CAC can now be made, and treatment can be tailored specifically to each patient.

Each of the following imaging sections will provide an overview of the specific technique and how calcium is identified, how CAC characteristics are quantified and to what degree, the corresponding CAC scoring system if available and how it is used to delineate which lesions are amendable to calcium modification or not and how the imaging technique can be used to judge if successful modification has occurred or not, unique applications and future technological advancements, and lastly, the modality's weaknesses.

## Coronary angiography

**Figure 2:**
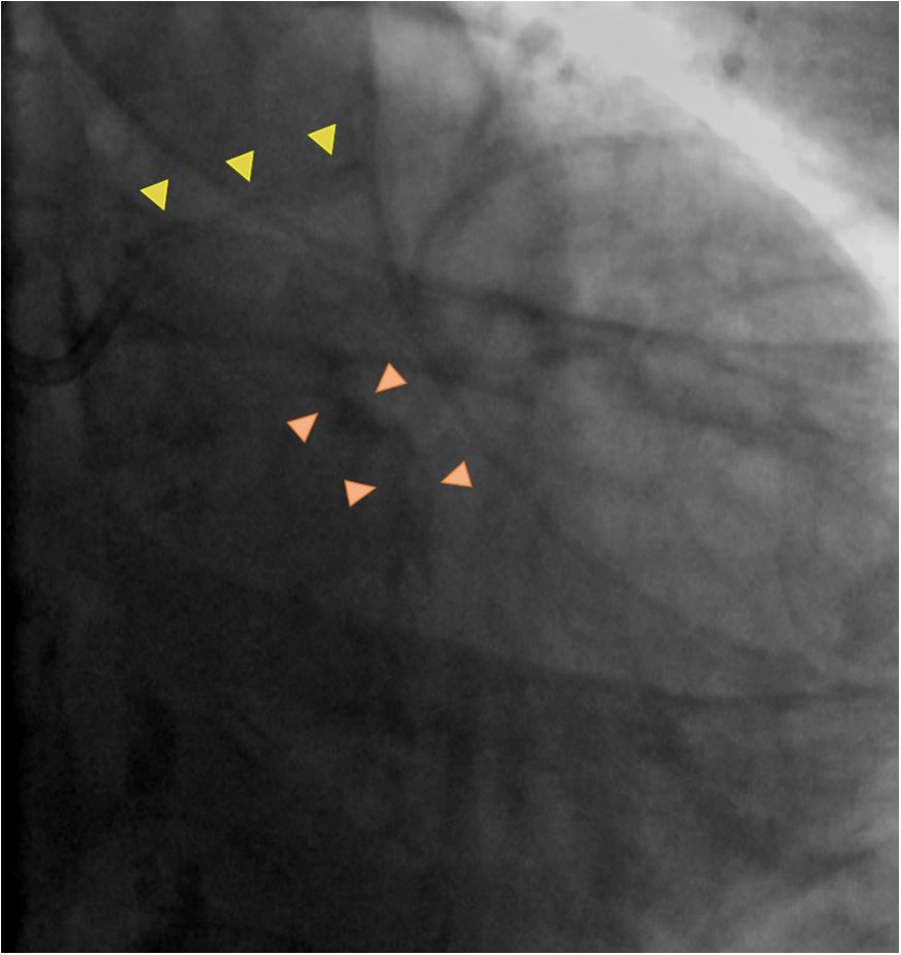
Coronary angiography calcification pattern summary. Calcium appears as gray or black outlines, easily assessable when no contrast dye is injected and which follow the coronary artery silhouette, with position excursion in line with vessel movement during the cardiac cycle. Mild calcification is defined as appearance of the calcified component only on one side of the vessel (yellow arrowheads), whilst severe calcification is defined as appearance of the calcified component on both sides of the vessel.

Coronary angiography has historically been the first modality used to detect CAC. On angiogram, CAC can be visualized as areas of linear x-ray attenuation along the course of a coronary artery (Figure 2). Angiography presents good specificity for CAC, however, in Mintz et al., coronary angiography had a diagnostic accuracy of about 38% (with 26% of those lesions having moderate CAC and 12% having severe CAC), which was corroborated in a more recent study by Wang et al. indicating an accuracy for coronary angiography of about 40.2% when compared to IVUS (8, 10). When assessing the ability of angiography to detect CAC patterns, the diagnostic accuracy increases when calcium eccentricity is >180°, length is >6 millimeters (mm), and superficial calcification is present, and accordingly, with lesser degrees of eccentricity, lower calcification length, and presence of deep calcium, accuracy falls to below 50% (8).

**Figure 3:**
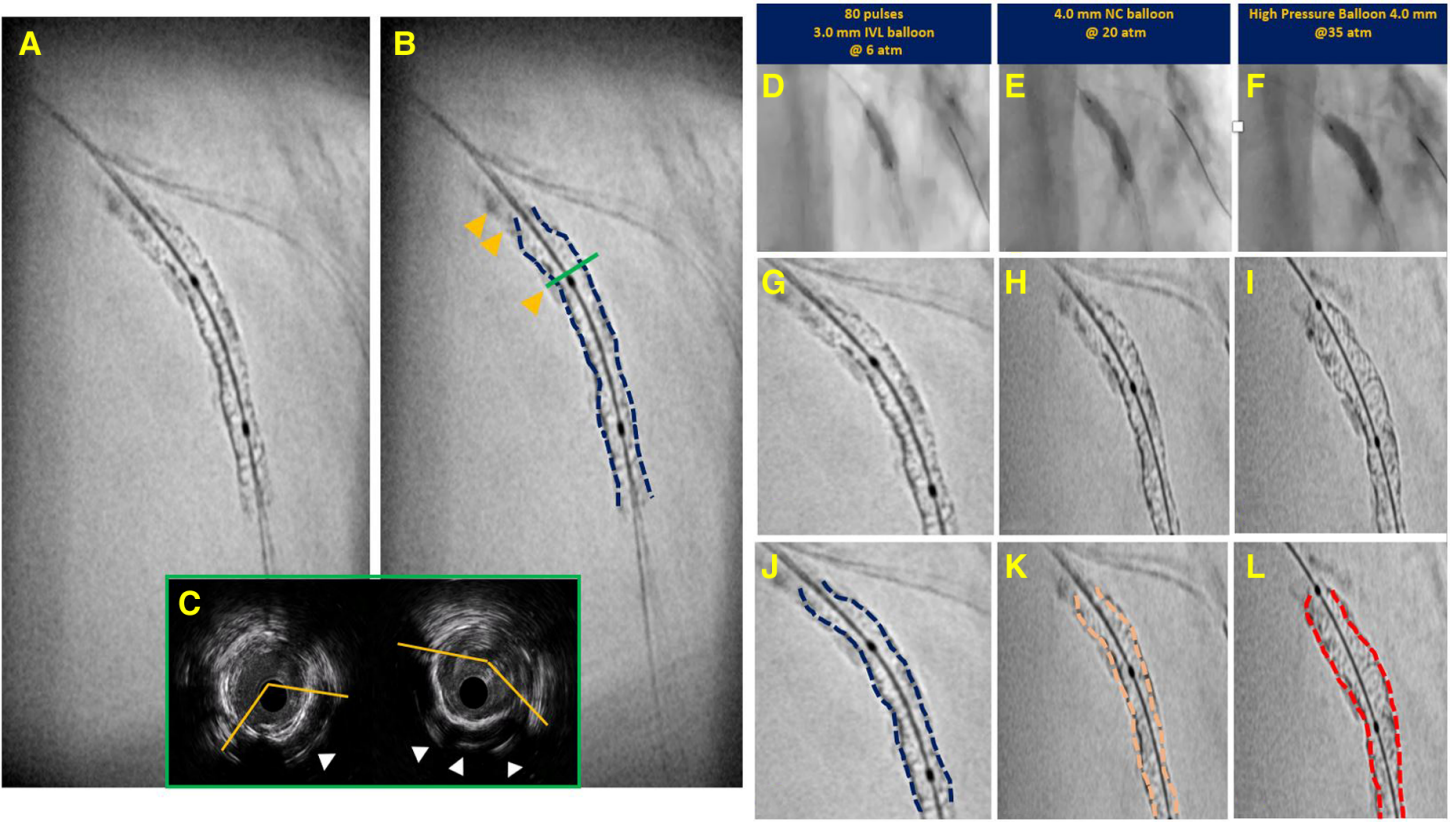
Case example of calcified in stent restenosis. Enhanced angiography (Panel **A**) proved helpful in defining presence of significant calcified component (yellow arrowhead in Panel **B**) and associated stent underexpansion (dotted blue line). On Intravascular ultrasound (at the level of green line in Panel **C**), calcium component appeared not circumferential and possibly thin according to evidence of reverberations (white arrowheads), even though they could have been misinterpreted for stent struts). Panels **D,E** show resistant calcium with underexpansion of intravascular lithotripsy (IVL) and non-compliant (NC) balloons. Eventually expansion achieved with high pressure balloon (Panel **F**). Comparing enhanced angiography at each step (baseline—Panel **G–J**; after lithotripsy and NC dilation in Panels **H–K**; after high pressure balloon in Panels **I–L**) it is possible to appreciate degree of calcium modification with increasing improvement of stent expansion.

Newer technology such as enhanced angiographic techniques (ClearStent (Siemens) or Stentboost (Philips)) can assist in guiding decision-making in cases of complex scenarios or in those circumstances where intravascular imaging modalities are unavailable and can give greater insight about calcium burden and symmetry as well as stent expansion. However, enhanced angiography still cannot provide a highly detailed illustration of calcification patterns, and its use comes with the cost of increased radiation exposure. As highlighted in Figure 3, enhanced angiography can potentially play a role in calcified in-stent restenosis, where the presence of multiple stent struts might interfere with the ability of intravascular imaging to image the deeper layers of the vessel wall with the risk of underestimating the degree of calcified component concealed behind the shadowing produced by stent struts.

As such, conventional angiography may be used to gauge the presence of significant CAC, but it does not allow for the definition of specific patterns of calcification or a determination on whether CAC has been successfully modified. Furthermore, the interpretation of CAC by angiography is limited by its reproducibility. In an analysis of the BioFLOW studies, intraobserver variability among peripheral centers as compared to a central core-laboratory analysis fluctuated by as much as 73%–79% in calcified lesions defined as moderate or severe, respectively (11). Given this, newer imaging modalities can be used in tandem with conventional angiography to supplement these weaknesses.

## Intravascular ultrasound

**Figure 4:**
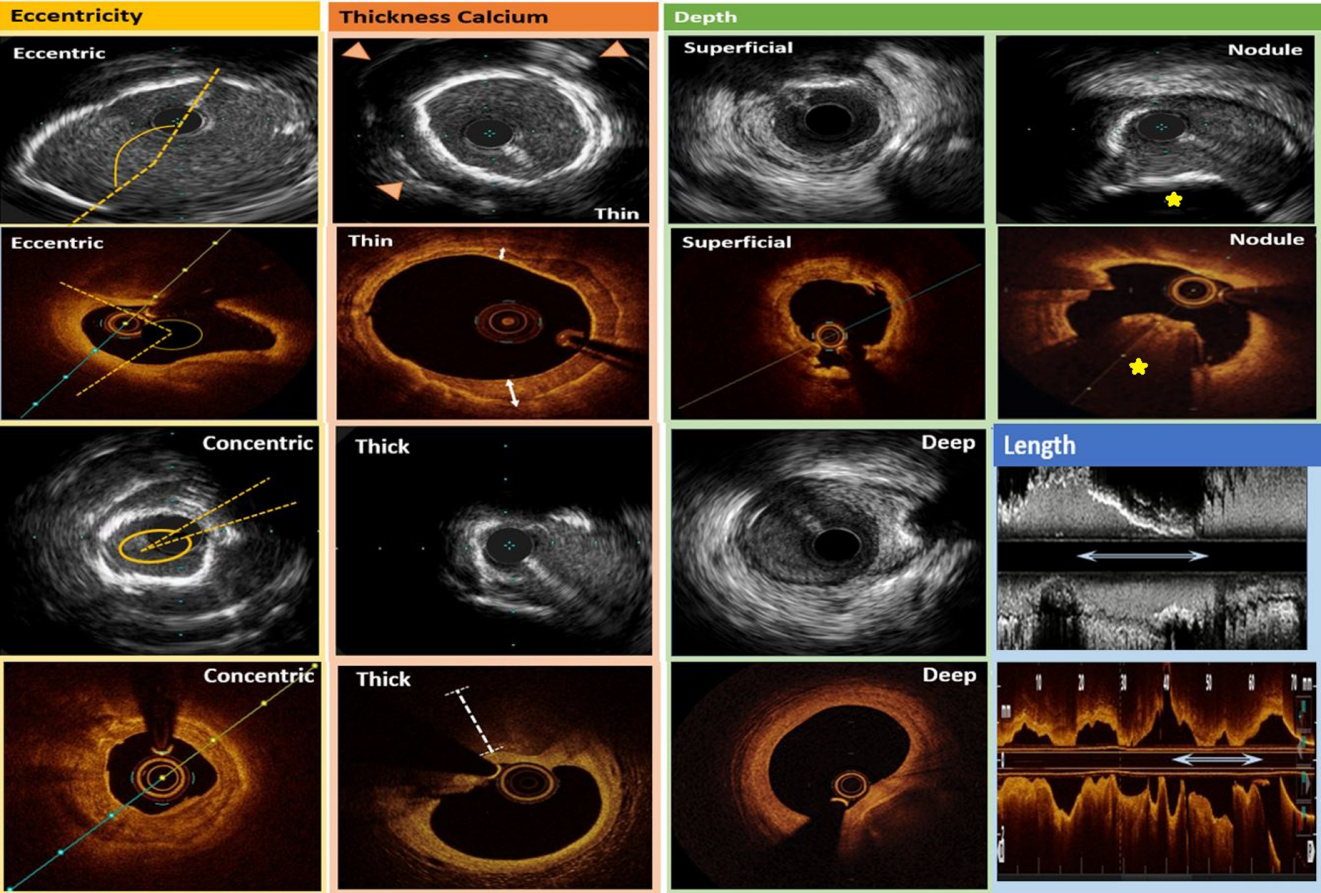
Intravascular ultrasound (rows 1 and 3) and optical coherence tomography (rows 2 and 4) calcification pattern summary. In the first column, calcium eccentricity is denoted by the dotted yellows lines which approximate the edges of the calcification. In the second column, thin calcium on IVUS is denoted by the presence of reverberation artifact (arrowheads) whereas thick calcium shows signal drop-out beyond the calcification. Also in the second column, thin calcium on OCT is demonstrated by the sharply delineated plaque with signal attenuation within its borders (bidirectional white arrows), and thick calcium on OCT is demonstrated by the white dotted line which shows signal drop-out beyond the calcium. In the third column, superficial and deep calcium intraluminal to the media or extraluminal to the media, respectively, can be visualized on IVUS and OCT. Lastly, in the fourth column, calcified nodules are denoted as yellow stars, and calcium length is illustrated by longitudinal bidirectional white arrows in both the IVUS and OCT examples.

CAC on IVUS imaging appears as areas of bright highly echo-dense (hyperechogenic) structure within the vessel wall (intima and/or media) and with posterior shadowing or signal drop-out. While IVUS was previously thought of as limited in its ability to measure calcium thickness because of signal-drop out beyond the calcium, recent studies have illustrated that IVUS can assess this variable with fair discrimination. The presence of a smooth calcium border with reverberation artifact beyond the calcification has been associated with a calcium thickness of less than 0.5 mm in 54.6% of cases whereas an irregular border without reverberation artifact within the region of signal-drop-out was associated with a thickness of greater than 0.5 mm in 75.9% of cases (10). In this way, IVUS can assess the presence and pattern of CAC with an overall diagnostic accuracy of 82.7%, which is comparable to a prior histologic study of 50 lesions (10, 12). The appearance of calcium on IVUS and a summary of calcification patterns is presented in Figure 4.

IVUS has a number of applications in CAC evaluation and intervention, and it can guide calcium modification therapy as well as stent deployment. First, an IVUS-based calcium scoring system referred to as the Calcium LADEN score has been proposed to predict which lesions may be amendable to calcium modifying procedures in order to optimize stent expansion (13). Summarized, if IVUS imaging shows calcification comprising over 270° of the wall circumference with two or more of the following criteria met including: over 270° of circumferential calcium in over 5 mm of vessel length, full 360° of circumferential calcium, calcified nodule, or vessel diameter under 3.5 mm, then calcium modification (rotational atherectomy according to the authors) should be considered. Additionally, after calcium modification, reassessment with IVUS and visualization of the previously described reverberation artifact or fissures/cracks within the calcified component can indicate adequate lesion preparation and modification of the calcium burden (10, 14). Lastly, IVUS-guided PCI for all lesion subtypes, including calcified, has translated into substantial clinical benefit. A recent meta-analysis of 27,610 patients demonstrated that those who underwent IVUS-guided PCI group experienced a relative risk reduction of 33% cardiovascular death as compared to those who underwent angiography-guided PCI, which is presumed related to the superior ability of IVUS to characterize vessel anatomy and atherosclerotic burden, optimize stent sizing, and avoid malapposition or underexpansion (15). Notably, IVUS may also assist in predicting, and thus avoiding, procedural complications such as the no- or slow-reflow phenomenon. Lesions associated with CAC extending >24 mm, an increase in the number of reverberations post-modification, and an arc of calcium at the minimum lumen area >300° were associated with increased rates of slow/no reflow and may alert physicians as to which patients may be at higher risk and might require preventative measures (16).

While IVUS offers multiple advantages when compared to coronary angiography, it presents a few limitations that are worthwhile to discuss. Most importantly, an accurate measure of CAC thickness in calcified lesions cannot be made, though can be inferred as previously discussed, which as a corollary means that an accurate assessment of the whole volume of calcium is not possible with IVUS. In addition, as calcium limits ultrasound penetration, characterization of tissue deep and behind the calcified component cannot be made. This same principle applies in the assessment of tissue and calcified burden in the presence of multiple layers of stent struts such as in calcified in-stent restenosis. Lastly, IVUS has lower accuracy for detecting microcalcifications (<50 micrometers) due to being below the spatial resolution threshold, which are more commonly associated with acute coronary syndrome (17).

## Optical coherence tomography

CAC on OCT imaging appears as sharply demarcated areas with signal attenuation within the lesion boundaries. The ability to define the abluminal border is the distinctive feature in differentiating a calcified atheroma from a lipidic one. As in IVUS, OCT is similar in that it enables the physician to assess eccentricity and longitudinal extension of CAC. However, OCT has an improved capacity to measure CAC thickness, though owing to lower tissue penetration of infrared light compared to ultrasound, OCT has a reduced ability to detect deep calcium with a limit to within about 1.5 mm of the vessel wall. This becomes even more apparent when the calcified component is located behind a lipidic pool as this causes signal drop-out. This accounts for why in a comparative study of OCT vs. IVUS in defining the presence of CAC, OCT was found to have a slightly lower accuracy (76.8%) vs. IVUS (82.7%) (10, 18). OCT calcification patterns are summarized in Figure 4.

OCT is more accurate in characterizing the various components of an atherosclerotic plaque, and specifically calcium, as it is the only technique that allows for a true volumetric quantification, derived from greater accuracy in defining calcium thickness and longitudinal extension. This is relevant as three-dimensional calcium volume has been proven to be a highly reliable predictor of balloon expansion at predilation and of subsequent adequate stent expansion (19, 20). Furthermore, an OCT-based calcium scoring system has been introduced to determine which CAC should undergo modification prior to stenting, similar to that of IVUS. The scoring system developed by Fujino et al. and referred to as the “5–5–5” rule is based on assigning two points for detection of calcium arc >180°, one point for calcium thickness >0.5 mm, and one point for longitudinal calcium extension >5.0 mm. A score of four is associated with a higher risk of stent underexpansion (96% vs. 78%) defined as minimum stent area <70% of mean reference area, and consequently, would call for additional lesion preparation (21). Moreover, OCT-derived features of CAC pattern can aid in predicting the response to calcium-modifying techniques as thinner (<0.67 mm) and more concentric (arch >227°) CAC were more likely to be associated with calcium cracking after modification (22). In this regard, due to its higher spatial resolution, OCT can detect, better than any other imaging modality, a reduction in calcium volume and improvement in lumen gain as well as occurrence of fractures after application of calcium-modifying techniques (23).

A practical example of the additive value of OCT guidance vs. IVUS guidance in addressing CAC has recently come from a retrospective analysis by Kobayashi et al. of 247 calcified lesions in which it was observed that OCT-guided rotational atherectomy was associated with a greater degree of stent expansion than IVUS-guided rotational atherectomy. A plausible explanation for this result was represented by a trend for larger burr size and more frequent burr-upsizing in the OCT arm presumably due to the higher resolution of OCT to define whether the CAC had been modified or not. However, whether the stent expansion achieved with OCT over IVUS translates into a clinical benefit was not demonstrated. Specifically in their work, Kobayashi et al. did not report a difference in target-lesion revascularization at one-year follow-up between IVUS and OCT guidance (24). This echoes evidence from large clinical trials such as the OPINION and ILUMIEN III studies. In both, despite not being specifically designed for the treatment of calcified lesions, OCT-guided PCI was non-inferior to IVUS-guided PCI in terms of achieved minimum stent areas, procedural success, and long-term rate of target vessel failure at 12 months follow-up (25, 26).

**Figure 5:**
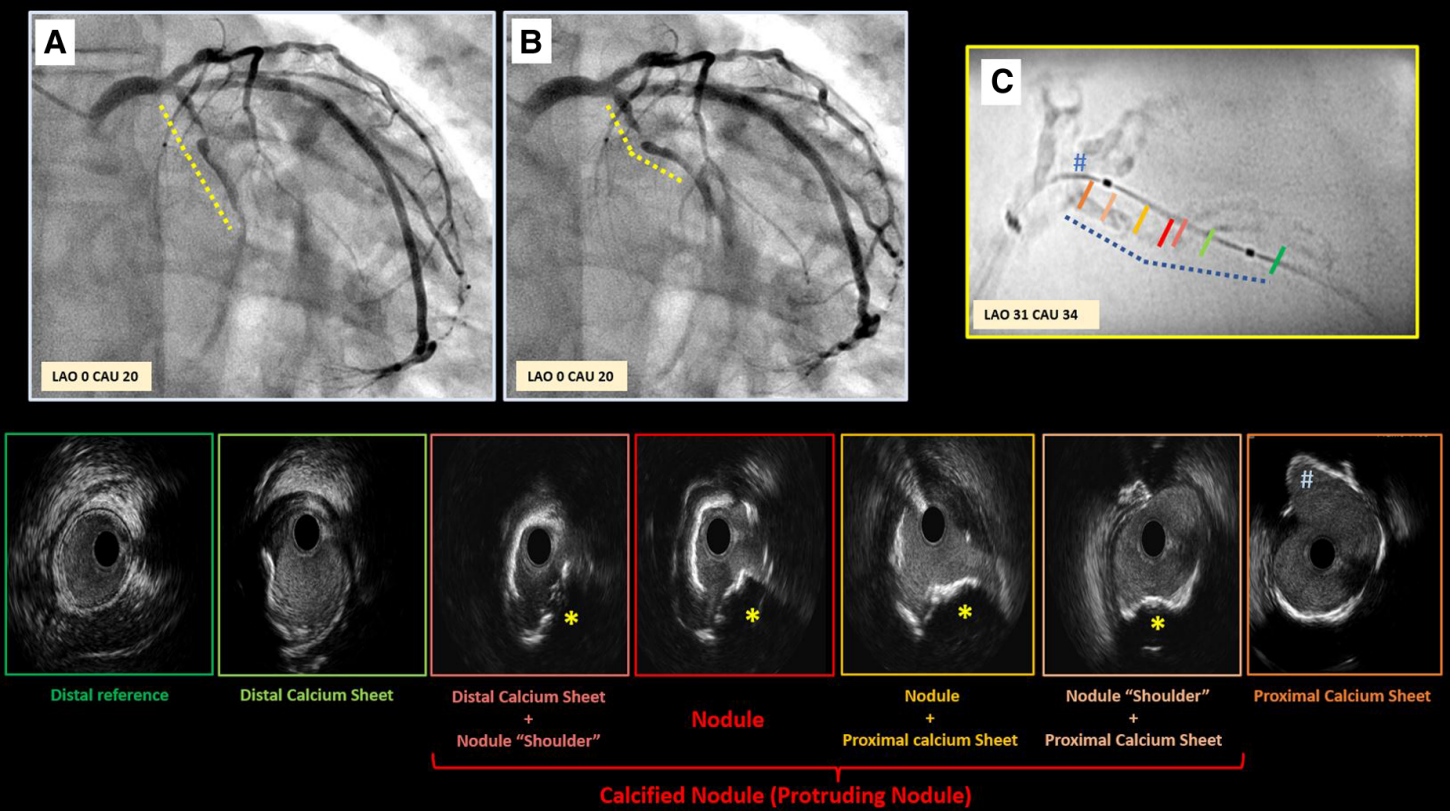
Case example supporting pathogenesis of calcified nodule. Panel **A,B** depict critical stenosis in left circumflex, with dotted lines highlighting the high motion and excursion of the mid part of the vessel compared to proximal segment during the cardiac cycle (Panel **A** exhibits diastole; **B** exhibits systole) with a hinge point right at the site of focal critical stenosis. Enhanced angiography clearly depicts the significant amount of calcium (Panel **C**). Intravascular ultrasound was performed revealing the presence of a non-concentric sheet of calcium distally that become more concentric as it gets closer to the focal stenosis (red panel) where a protruding nodule is clearly detectable (yellow star). This calcified sheet continues proximally into a non-concentric sheet of thick calcium as approaching the ostium of the first marginal branch (#).

A peculiar pattern of CAC where OCT shows unique diagnostic accuracy over any other imaging modality is represented by calcified nodules, which is a pattern of extremely eccentric and thick calcium with eventual protrusion into the lumen. This pattern of CAC is gaining increasing attention for its prognostic implications as it accounts for roughly 5% of plaque instability in acute coronary syndrome with the remaining 65% and 30% explained by mechanisms of plaque rupture and plaque erosion, respectively (27). Recent histopathological analysis has shown that calcified nodules are typically located at sites of the coronary artery subject to high motion/torsion (“hinge points”) during cardiac cycle and more frequently at the level of the right coronary artery (61%) and at its mid-segment (56%) (28). An example of a large, calcified nodule at a hinge point in the left circumflex artery with cross-sectional intravascular images is provided in Figure 5. OCT, like IVUS, shows a particular accuracy to detect this pattern of CAC, however, OCT is the only modality offering the extra benefit to truly differentiate the two main patterns of calcified nodules, namely protruding vs. eruptive. The former is featured by a calcified nodule protruding towards the lumen and covered by a smooth and regular-appearing fibrous cap, while the latter appears as a nodule with an irregular surface associated with a disrupted cap and possibly thrombotic material. This distinction has prognostic implications as eruptive nodules have been consistently associated with worse clinical outcome in terms of cardiac death and target lesion failure (29, 30). However, despite being associated with a better prognosis, protruding nodules more frequently lead to suboptimal stent expansion and lower minimal stent area after PCI when compared with eruptive nodules suggesting that they affect overall vessel compliance more than their eruptive counterpart (30). This observation might have practical implications as the optimal approach to address this pattern of CAC remains unclear with a substantial paucity of evidence to support the use of one technique over another. While orbital atherectomy and rotational atherectomy are advocated for as first line strategies to treat calcified nodules because of their debulking potential, data has not demonstrated any benefit, though promising preliminary results seem to come from the application of intravascular lithotripsy (31, 32).

**Figure 6:**
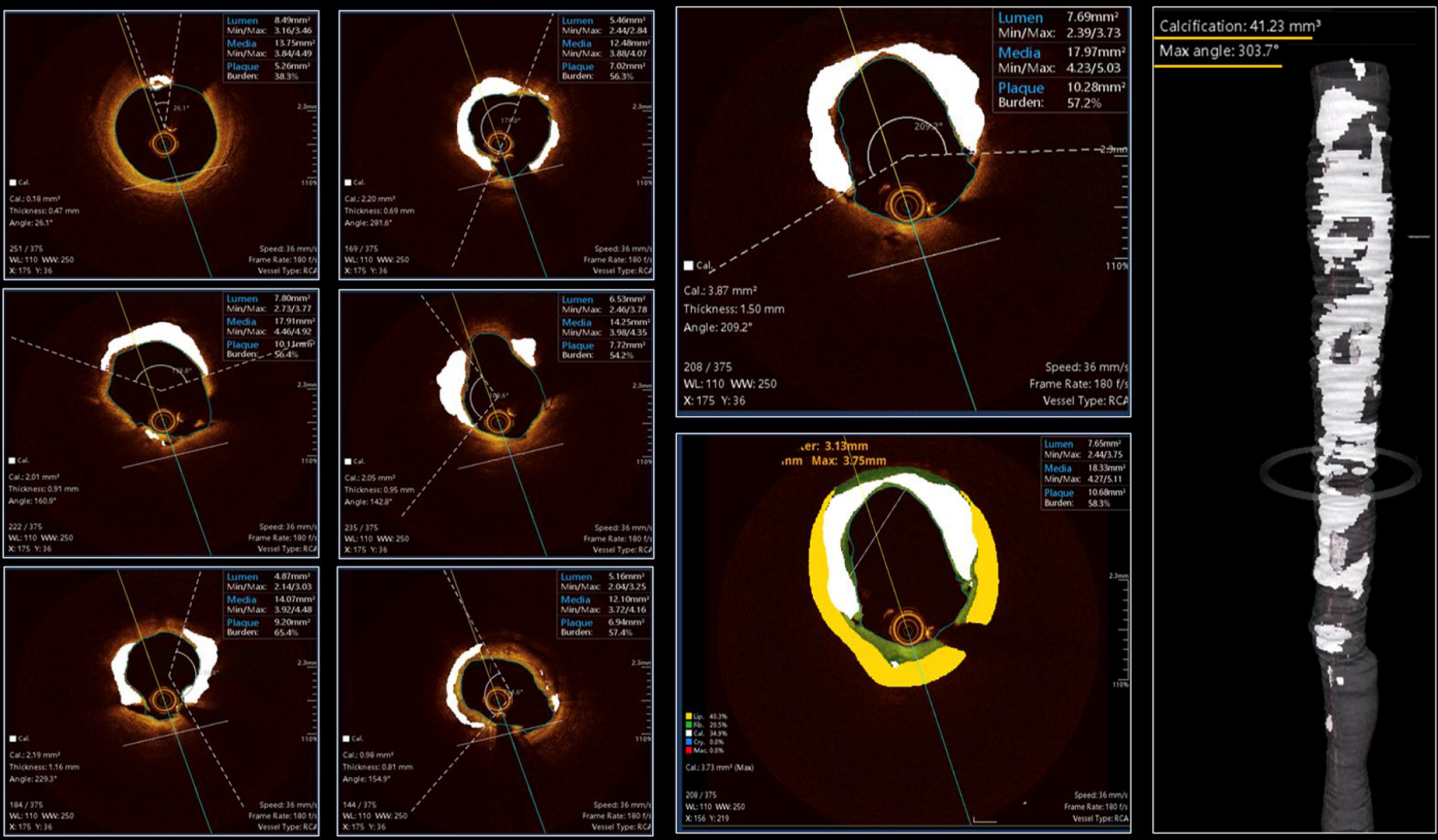
Case example applying OCT-plus package for automated detection of calcium burden and distribution. The six panels on the left provide insights about all features of OCT-patterns of calcium. The large two panels in the middle, provide high detailed image of calcium distribution and its possible combination with non-calcified tissues [lipidic (yellow) or fibrotic (green)]. The reconstruction on the right provide clear overview of longitudinal distribution of calcium and provide details about the maximum angle of calcium and total calcium volume in mm^3^.

**Figure 7:**
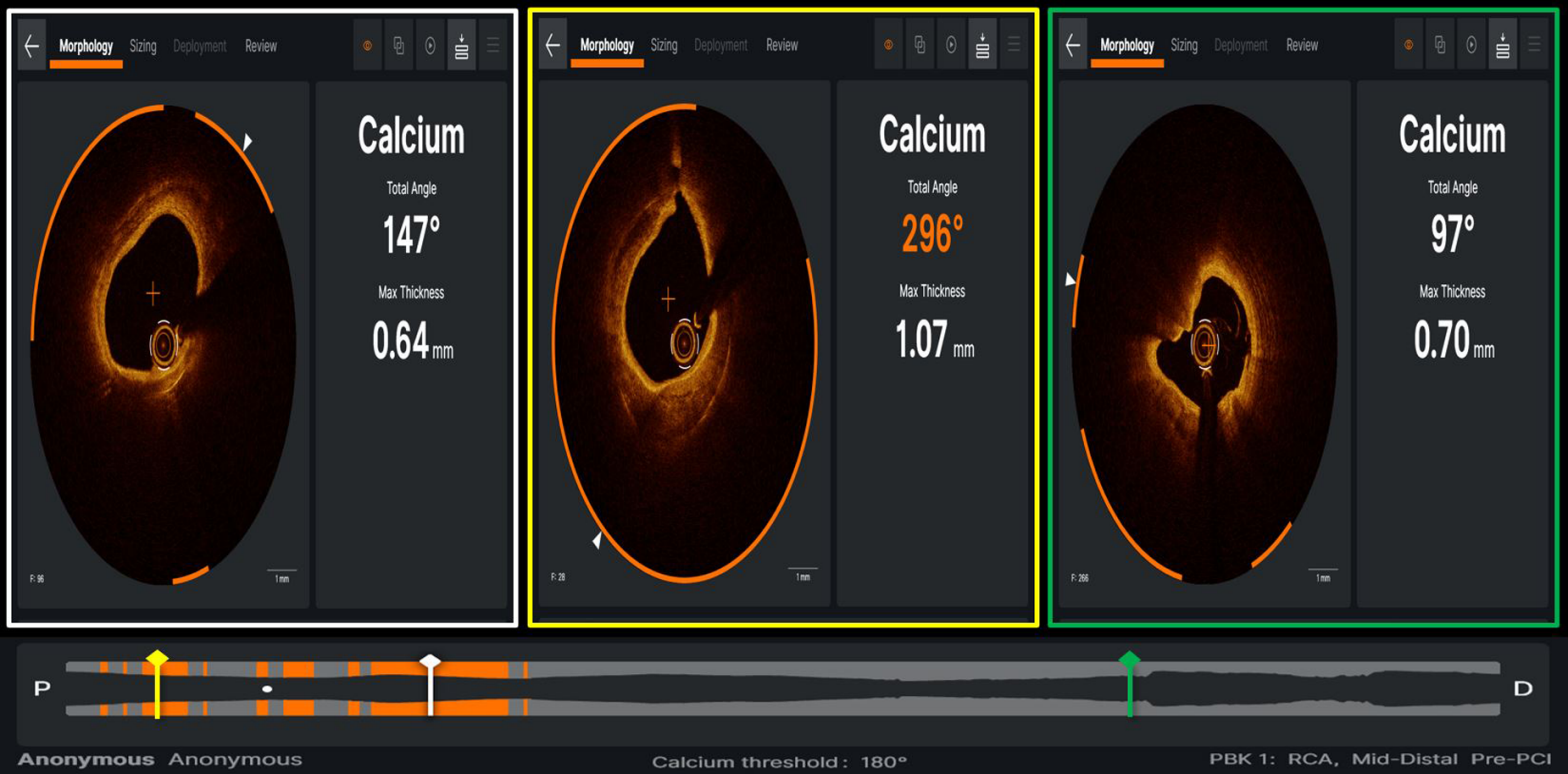
Case example of optical coherence tomography applying ultreon software (Abbott) for automated detection of calcium burden and distribution, expressed by calcium arc (orange arcs in the three panels) and maximal thickness (defined by white arrowhead on each OCT cross-section).

One of the most recent advancements in OCT technology is the availability of artificial intelligence (AI)-based software (Ultreon 1.0 Software, Abbott, US; OCT-Plus, Pulse Medical, China), which is able to automatically select and characterize CAC in a real-time and fully automated fashion with the additional benefit of removing an operator's interpretation bias. In a proof-of-concept study including 10,517 training set images and 1,156 testing set images, an AI-based approach was able to detect CAC with an accuracy of 88.5% (33). An example of the OCT-Plus software interface is provided in Figure 6 and that of Ultreon is provided in Figure 7.

While it is true that OCT provides higher detailed characterization of CAC, some limitations should also be acknowledged. OCT is limited in its ability to assess deep CAC especially when behind a lipidic or necrotic core (34). Additionally, due to suboptimal blood-clearance of the lumen, OCT is limited in assessing CAC in the context of very large caliber/ectatic vessels or in cases of aortic-ostial lesions, all settings where IVUS has an indisputable advantage.

## Coronary computed tomography angiography

Coronary computed tomography angiography has assumed a larger role in the diagnostic pathway of patients with coronary artery disease. Calcification on CCTA appears as bright areas with Hounsfield units >130 due to significant x-ray attenuation and within a typical coronary distribution. Mainly in view of its excellent negative predictive value, it has become a first line tool to rule out obstructive coronary disease, and CCTA offers a non-invasive approach to identify CAC with an accuracy of 94% (35). CCTA can also provide insights about calcium eccentricity and longitudinal extension to a similar extent as OCT. However, due to blooming artifact, when it comes to actual calcium burden quantification, it has been shown that compared to OCT, CCTA can overestimate calcium volume by 60%, and it is unable to determine calcium thickness. Overestimation appears to proportionally increase with increasing calcium burden (36). As such, CCTA cannot distinguish between nodular and non-nodular calcifications.

Nevertheless, similar to IVUS and OCT, a calcium grading system using CCTA has been shown to be a predictor of which lesions were most likely to undergo rotational atherectomy during intervention. In a retrospective study including 241 moderately or severely calcified lesions, Yu et al. demonstrated that while calcium eccentricity was a significant predictor of which lesions would undergo calcium modification during subsequent intervention, their novel calcification remodeling index calculated as the ratio of the smallest cross-sectional area of the lesion to the proximal reference luminal area was the most likely predictor (37). Of note, calcification length was nearly a significant predictor of modification at *p* = 0.053.

With improvements in technology and subsequent spatial resolution, CCTA-based characterization of CAC may be further improved. In a sub-analysis of a study comparing coronary plaque characterization by IVUS to conventional CCTA and a new-generation whole-heart coverage CCTA using 256 slices, the mean difference in calcified plaque volume between IVUS and the 256-slice CCTA was less than that between IVUS and the conventional CCTA (38). Moreover, with newer generation computed tomography scanners and de-blooming computer software, the effect of blooming artifact can be mitigated though not completely eliminated (39). And lastly, CCTAs with submillisievert levels of radiation are now available and represent an advancement in lowering the dosage of radiation patients receive when undergoing a scan without compromising image quality (40).

**Figure 8:**
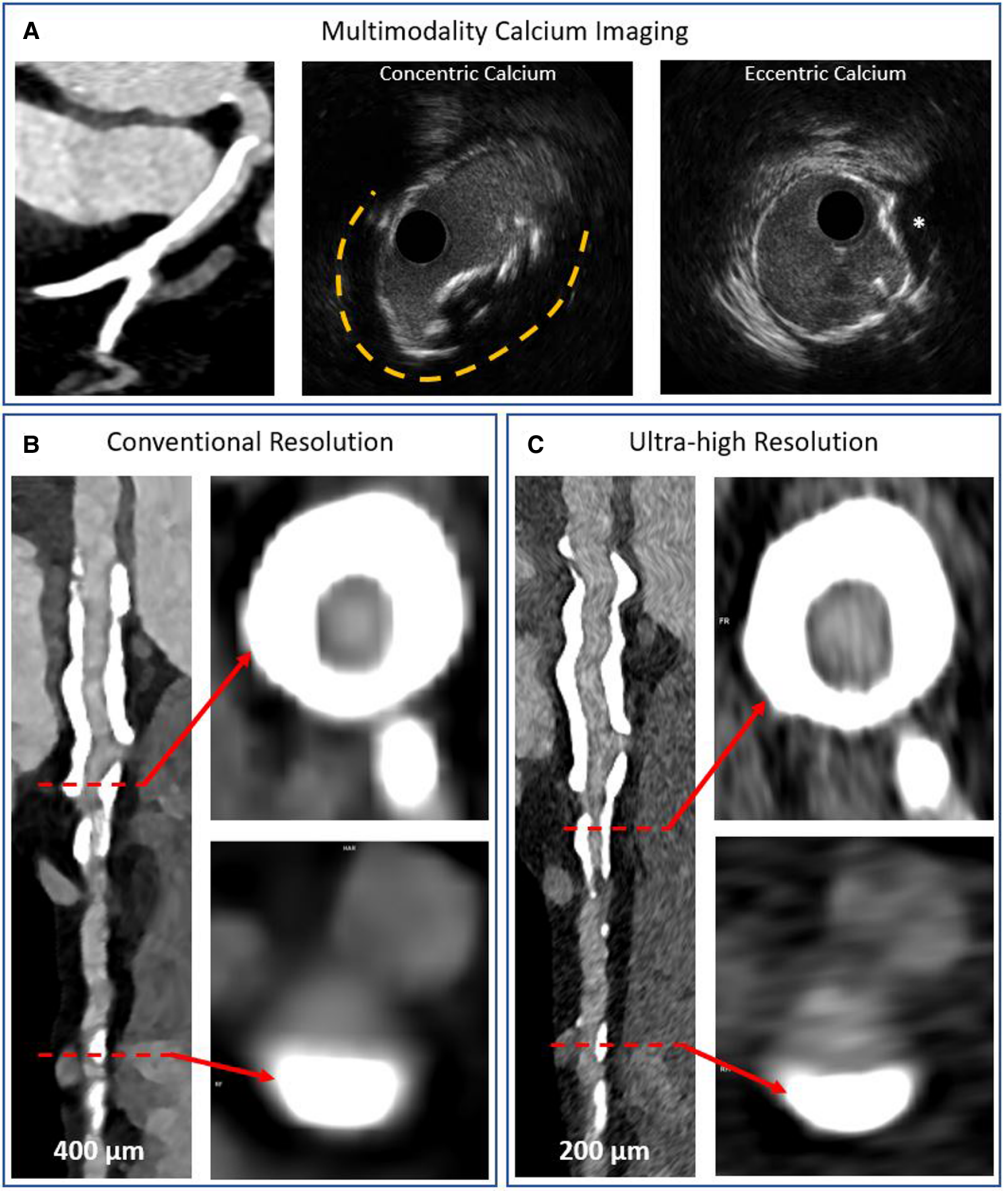
Multimodality imaging of calcified coronary arteries. Panel **A** shows a heavily calcified coronary artery segment on conventional detector CT imaging and intravascular ultrasound imaging examples of concentric and eccentric calcification patterns. The asterisk denotes a calcium nodule. Panels **B,C** show photon counting CT acquisitions of a heavily calcified vessel with concentric and eccentric calcification patterns at conventional and ultra-high resolutions, respectively. The blooming artefact is reduced while the lumen is more clearly visualized in the ultra-high resolution acquisition.

A further step forward in CCTA-based characterization of CAC might come from the recent introduction of photon-counting (PC) detector technology in CT scanning. PC CT imaging offers high temporal (66 milliseconds) and spatial (130 micrometers) resolution and multi-energy acquisitions that have improved its spectral resolution (41). The anticipated improvement in feature detection and material quantification will allow the selective isolation of different materials (e.g., calcium, iodinated contrast) and lead to more accurate calcium characterization (thickness), reduction of blooming artifacts, and superior luminal stenosis evaluation (42, 43). The first imaging systems have been installed and although early-stage, clinical experience is promising (Figure 8).

In this regard, there has been a new approach in planning PCI by creating a CCTA-derived 3-dimensional reconstruction of the coronary vasculature to provide a “road-map” in order to facilitate intervention. Guiding catheter selection, optimizing angles for angiographic views, and quantifying plaque burden and composition can all be derived or inferred from CCTA, allowing a full pre-planning of coronary intervention, mimicking the same approach already seen in structural interventions (44).

At this time, CCTA is valuable in providing an overview of calcium burden and distribution by giving the operator an indication of whether advanced lesion preparation is required or not with positive implications in terms of planning the catheterization laboratory's workflow and of patient understanding what the procedure will entail at the time of consenting. However, until more supporting evidence becomes available, CCTA is unable to determine which modifying techniques are suitable for a particular lesion. In this regard, unlike intravascular imaging modalities, there are no CCTA-based scoring systems available to predict which CAC may undergo successful calcium modification. Moreover, CCTA cannot provide insights about degree of CAC modification meaning that it can be used for procedure planning but not for intraprocedural guidance.

## Integrating imaging results into the interventional approach

**Figure 9:**
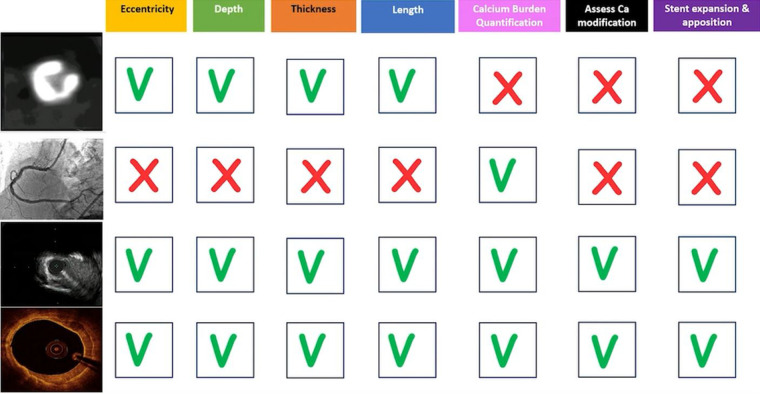
Summary of coronary artery calcification patterns and which imaging modalities are best suit to quantify each variable as well as which modalities may be used intraprocedurally to assess stent placement.

Detection and characterization of CAC using one or a combination of the imaging modalities previously discussed (Figure 9) has the ultimate aim of selecting the most appropriate calcium modification technique to most effectively address a specific pattern of CAC. This is crucial as a variety of calcium modifying therapies are now available to improve vessel compliance and optimize stent placement. Explaining in detail the techniques available for each CAC pattern is beyond the scope of this review, though we will instead briefly describe the mode of action of each technique in order to clarify which pattern of CAC each technique is more likely to be effective on.

**Figure 10:**
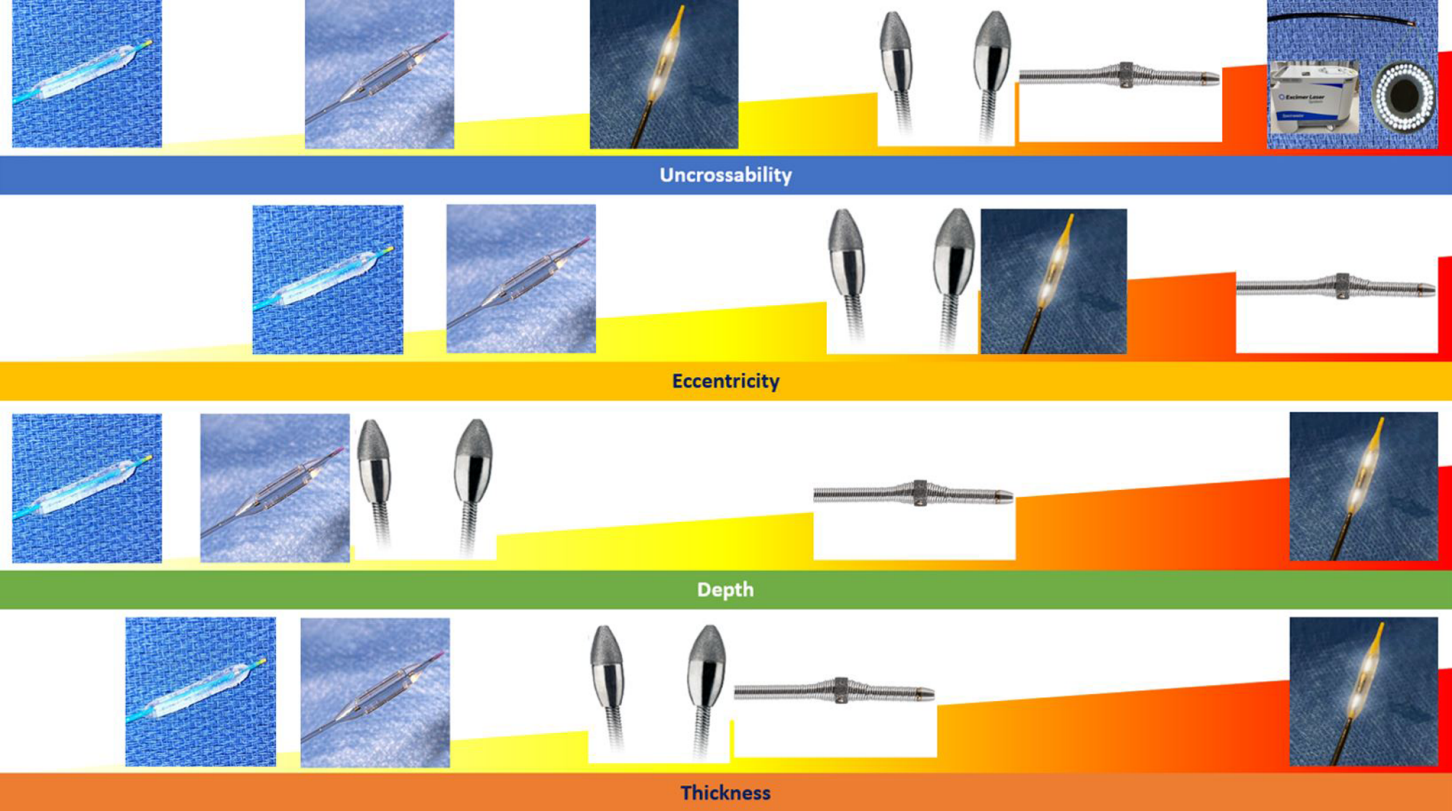
Summary of coronary artery calcification modification tools and most appropriate calcification patterns for their use.

Calcium-modification techniques can generally be divided into debulking techniques including rotational atherectomy, orbital atherectomy, and excimer laser as well as non-debulking techniques (also referred to as “balloon-based techniques”) including cutting balloons, scoring balloons, super high-noncompliant balloons, and lithotripsy balloons (Table 1, Figure 10).

**Table 1:** Summary of coronary artery calcification lesion modification tools.

	Ablation techniques			Balloon-based techniques			
	Rotational atherectomy	Orbital atherectomy	Excimer laser	Cutting balloon	Scoring balloon	Super high-compliant balloon	Lithotripsy balloon
Technology	High-speed rotating diamond-tipped burr	High-speed rotating diamond-coated crown	Ultraviolet light pulsations	Balloon mounted with longitudinal microblades	Balloon wrapped with wires/scoring element	Twin-layered noncompliant balloon	Balloon mounted with pulsed energy emitters
Mechanism	Differential abrasion	Differential abrasion	Photoablation *via*: • Photochemical • Photothermal • Photomechanical	Plaque cut	Plaque fracture	Displace calcium without modifying it	Calcium cracking *via*: • Compression • Shearing • Spallation • Squeezing • Cavitation
Size/Catheter compatibility	• 1.25 to 2.5 mm burr • 6 to 10 French	• 1.25 mm crown • 6 French	• 0.9 to 2.0 mm • 6 to 8 French	• 2.75 to 3.5 mm • 6 French	• 2.0 to 3.5 mm • 6 French	• 1.5 to 4.5 mm • 6 French	• 2.5 to 4 mm • 6 French
Applications	• Uncrossable lesions • Nodular calcium • Concentric calcium • Superficial calcium	• Uncrossable lesions • Nodular calcium • Eccentric calcium • Superficial calcium	• Uncrossable lesions • Calcified undilatable or uncrossable ISR	• Superficial calcium • Thin calcium • In-stent restenosis	• Superficial calcium • Thin calcium • In-stent restenosis	• Stent optimization • Refractory undilatable calcium especially within stent	• Deep calcium • Thick calcium • Eccentric calcium (?) • Nodular calcium (?)
Risks	• Perforation • Dissection • burr lodging • slow/no reflow • Bradycardia/AV block	• Perforation • Dissection • slow/no reflow	• Perforation • Dissection	• Dissection	• Dissection	• Perforation • Dissection	• Perforation • Dissection • Ectopics/Capture on ECG
Other	Eccentric Calcium *via* • Wire biasing • Multiaxial Rotablation (MAX)	• Superior in large caliber/aneurysmatic vessel • Can ablate in forward and backward directions • Low risk of crown lodging	• Requires continuous saline flushing • Flushing with contrast to potential photomechanical effect for calcified ISR	• Large profile, preventing crossing calcified disease • Limited action on large calcium burden	• Limited action on large calcium burden	• Unpredictable plaque behaviour at high @atm inflation • Safer for undilatable calcified ISR	• Large profile, preventing crossing calcified disease • 80 pulses per catheter

## Rotational atherectomy

Rotational atherectomy (RA) (Rotablator and Rotawire system, Boston Scientific) utilizes a diamond-tipped burr mounted on a pressurized-gas powered drive shaft and acts to preferentially erode fibrocalcific plaque *via* the principle of differential cutting when passed anterogradely through the stenosis (45). The burr itself comes in different sizes from 1.25 to 2.5 mm with an optimal burr size to artery ratio of 0.5:0.6, and it is advanced over a 0.009-inch wire (RotaWire Floppy or RotaWire Extra Support). Of note, an updated system (RotaPro, Boston Scientific) is available with an enhanced user interface for improved operability.

In the only randomized study to date, the ROTAXUS trial, RA was found to be effective in achieving acute lumen gain, but at 2-year follow-up, there was no difference in cardiac outcomes (46, 47). In the PREPARE-CALC trial, this finding was corroborated with RA being superior to balloon-based CAC modifying techniques in achieving successful stent delivery, expansion with <20% in-stent residual stenosis, and Thrombolysis in Myocardial Infarction grade 3 flow, though with no difference in 9-month in-stent lumen loss and cardiac outcomes (48).

In view of its mode of action, RA finds its main application in uncrossable calcified lesions, and it exerts ablative action predominantly on superficial calcium rather than deep calcium. Because the rotaburr advances over a wire, the wire position might itself bias the passage of the rotaburr away from calcium in cases of eccentric CAC. This is why, theoretically, the more concentric the calcified pattern, the more RA can provide debulking power. In experienced hands, the action of RA can also be applied to eccentric calcium by addressing the wire-bias (e.g., moving the position of rotawire in side-branches until the passage of the rotaburr is biased towards the calcified component). Lastly, RA may provide debulking action on calcified nodules, although supporting evidence is limited and controversial (31). Potentially, multiaxial rotablation technique could be considered in highly experienced operators to debulk calcified nodules or very eccentric calcium. The technique consists of reducing rotaburr revolution speeds with consequent oscillatory movement of the rotaburr directed towards the more external part of the vessel (rather than following a linear trajectory moving antegradely), mimicking the behavior of the crown in orbital atherectomy (49).

## Orbital atherectomy

Orbital atherectomy (OA) (Diamondback 360 Coronary Orbital Atherectomy System and ViperWire, Cardiovascular Systems Incorporated) uses a diamond-coated crown eccentrically mounted on a pressurized-gas powered drive shaft and acts similarly to RA by preferentially ablating fibrocalcific plaque. The crown comes in one size (1.25 mm) so that it can suit every vessel dimension. The system uses a dedicated 0.014-inch wire (Viper-wire) with features similar (though not identical) to a conventional workhorse wire, making it deliverable to the distal segment of the treated vessel in most anatomies, offering an advantage over RA. An additional advantage offered by OA is the ability to ablate both anterogradely and retrogradely with technically no risk of crown entrapment.

For its mode of action, OA should be considered for uncrossable stenosis but also in cases of superficial, either eccentric or concentric, calcium especially in the context of large caliber vessels or highly tortuous vessels. There is currently one randomized study (ECLIPSE trial) evaluating the efficacy of CAC preparation by OA vs. conventional balloon angioplasty prior to DES delivery, however, the trial is ongoing and results are still pending (50). The non-randomized ORBIT I and ORBIT II trials have, however, largely established the safety, feasibility, and effectiveness of OA for CAC modification (51, 52).

## Excimer laser

The current excimer laser system (CVX-300 ELCA System, Philips) uses the photoablative ability of a xenon chloride laser to modify CAC by three mechanisms: photochemical (breaking of molecular bonds), photothermal (plaque modification by production of heat), and photomechanical (production of high-energy bubbles hitting and cracking the calcified plaque component). The device size ranges from 0.9 mm to 2.0 mm, which corresponds to the diameter of the tunnel created by advancing the laser-catheter through the atheroma.

Uncrossable lesions and undilatable stent in calcified restenosis represent the key indications for laser when applied to CAC. There are no randomized trials examining the effectiveness of the excimer laser system, but in a study of 126 uncrossable lesions, excimer laser use was successful in crossing 81.8%, 62.7% of which had moderate or severe calcification (53). In a study of 81 cases of in-stent restenosis, excimer laser use was also associated with more calcium fracturing and a larger final minimum lumen area as well as greater peri-stent calcium fracture noted when comparing pre- and post-modification OCT imaging (54).

## Balloon-based techniques

Balloon-based techniques are associated with modification of the calcified component include cutting balloons, scoring balloons, and intravascular lithotripsy balloon.

Cutting balloon technology consists of a non-compliant balloon with three or four sets of longitudinal microblades on the surface, so when the balloon is inflated, the blades produce superficial fissures within the CAC. It is generally reserved for eccentric, thin, and superficial CAC usually as an adjunct to more advanced techniques of calcium modification. In an early randomized study of 521 patients undergoing cutting balloon angioplasty or conventional balloon angioplasty before IVUS-guided stenting, the patients undergoing cutting balloon angioplasty had a larger minimal lumen area and lower rate of restenosis (55). This has been recently replicated in the COPS study (56).

Compared to cutting balloons, scoring balloon technology consists of a semi-compliant balloon with nitinol wire either parallel to the balloon or wrapped around it in a helical pattern as a means to more evenly disperse the fracture force upon inflation, preventing balloon-slippage, and reducing the risk of dissection or perforation (57). Though there are no randomized trials to compare the outcomes of scoring balloon application to CAC, scoring balloon use has been demonstrated to achieve larger minimum stent areas compared with pre-dilation alone (58, 59). They find similar application in CAC as cutting balloons and offer the advantage of lower profile and higher lesion-crossability.

## Lithotripsy balloon

The intravascular lithotripsy balloon (Shockwave Coronary Rx Lithotripsy System, Shockwave Medical) utilizes pulsatile mechanical energy at a frequency of one Hertz, which is released at the level of two emitters mounted on a semi-compliant balloon. As electrical current passes through the emitters, bubbles are generated within the contrast filling the balloon, generating a shockwave that is transmitted through the deeper layers of the vessel wall. As such, intravascular lithotripsy is potentially the only modality to modify thick and deep calcium though it can be used to address superficial, concentric or eccentric, and nodules as well.

There are currently no randomized trials available to compare the efficacy of intravascular lithotripsy in CAC against other modalities though in a pooled analysis of the DISRUPT CAD studies consisting of 628 patients of whom 97.0% had severe calcified disease, intravascular lithotripsy met the primary safety endpoint of freedom from major adverse cardiovascular events at 30 days in 92.7% of cases, and 92.4% met the primary effectiveness endpoint defined as stent delivery with a residual stenosis ≤30% (60).

## Conclusion

Percutaneous revascularization in patients with calcified coronary artery disease remains a persistent challenge. In this regard, understanding the pattern of calcification is critical as none of the available calcium-modification techniques can address all the different patterns of coronary calcification in the same manner and to the same degree. Multi-modality imaging allows the operator to define the pattern of CAC and develop a strategy for its modification. OCT and IVUS are indeed pivotal not only for procedural planning but also for fine-tuning the need for additional calcium modification intraprocedurally and, of course, for guiding final stent optimization. A significant contribution to the field will likely come from CCTA for use in procedural planning, especially as significant efforts have been made to mitigate the effect of the blooming artifact and improve spatial resolution.

The range of technologies and devices to address CAC has significantly grown over the last decade as well, and while this is of great value, it at the same time poses new challenges in terms of device selection. This calls for a clear approach that enables operators to select the optimal calcium-modifying technology at the right time and in the right context to guarantee a standardized approach among centers and operators.
